# Dirty Bread, Forced Feeding, and Tea Parties: the Uses and Abuses of Food in Nineteenth-Century Insane Asylums

**DOI:** 10.1007/s10912-019-09603-8

**Published:** 2020-01-07

**Authors:** Madeline Bourque Kearin

**Affiliations:** Anthropology Department, Brown University, Box 1921, Providence, RI 02912 USA

**Keywords:** Insanity, Asylums, Nineteenth century, Food

## Abstract

Nineteenth-century psychiatrists ascribed to a model of health that was predicated on the existence of objective and strictly defined laws of nature. The allegedly “natural” rules governing the production of consumption of food, however, were structured by a set of distinctively bourgeois moral values that demonized over-indulgence and intemperance, encouraged self-discipline and productivity, and treated gentility as an index of social worth. Accordingly, the asylum acted not only as a therapeutic instrument but also as a moral machine that was designed to remake lazy, indolent transgressors into useful, “decorous” citizens. Because the theory and mechanics underlying this machine seemed straightforward and self-evident to psychiatrists, they were confounded when the asylum failed to translate its ideals into reality. While psychiatrists tended to blame this failure on the intractable immorality and weakness of individual patients, particularly paupers and immigrants, a review of the various meanings and uses of food in the hospital reveals the fault lines that ran through the asylum’s ideological structure.

## Introduction

The nineteenth-century lunatic hospital formed the center of a new approach to madness known as moral treatment, which was first elaborated in France and England in the late eighteenth century and was subsequently imported into the United States (Scull 1981). Departing from earlier beliefs that lunatics were flawed beyond the hope of redemption, moral treatment emphasized their essential humanity, which remained intact despite their disease. The proponents of moral treatment emphasized “careful attention to cleanliness, exercise, air, and substantial diet” as a means of supporting the total health of the patient through the creation of a salutary environment (*Annual Report* 1833, 6). The belief that insanity was essentially a manifestation of temporary disturbances in the material support of the mind justified the conviction that a return to moderate habits and modes of living would reliably effect a cure in all but the most severe cases (Coventry 1846).

Michel Foucault describes how the rhetorical mechanics of moral treatment transmuted social disorder into individual pathologies, while middle-class mandates surrounding morality, productivity, and gentility were reinvented as modes of “treatment.” Foucault identifies this phenomenon as evidence that asylums were machines of “social control,” intended to “guarantee bourgeois morality a universality of fact and permit it to be imposed as a law upon all forms of insanity” (1971, 259). To David Rothman – also a proponent of the theory of social control – total institutions represent a solution to the emergent elite’s “profound uneasiness about the fragility of the social order,” designed to bring the disorderly in line with its distinctive vision of reality (2002, xl). More recently, historians have argued that such theories obfuscate the complex and dynamic nature of the asylum. Roy Porter, among others, has pointed to the “simplistic and over-generalized” aspects of social control theory and its failure to hold up when applied to a multiplicity of contexts (2002, 93). In *Madness: A Brief History,* Porter illustrates the wide variety of institutional experiences across place and time and how they confound attempts to generalize about the asylum (ibid., 93). Furthermore, he points to the “polyphonic nature” of the production of insanity, which consists not only of the diametrically opposed forces of psychiatrist and patient but also of a process of “complex bargaining between families, communities, local officials, magistrates, and superintendents” (99).

Porter’s work is illustrative — and to some extent was generative — of a shift in historiography beginning in the 1980s, representing both a dialogue with and a departure from the theory of social control (Porter 1985; Bivins and Pickstone 2007; Bynum and Shepherd 1985). This historiography targets the gaps and dissonances in the history of the asylum, taking as its object the disjuncture between the ideals of the asylum and the realities of practice, the alternating and overlapping practices of collusion and conflict between the asylum’s multiple actors, and the patterns of presence and absence that constitute the archive. While acknowledging that such absences make it difficult to access the experiences of patients, this new generation of scholars has nonetheless located sites where reservoirs of evidence may be found. They emphasize the need to take a critical perspective to the archive, recognizing its limitations as well as its potential. Recent research problematizes the “grand sociological theories” of Foucault’s generation and suggests a more balanced conception of the competing narratives surrounding the history of psychiatry (Luhrmann 2001, 4).

In this paper, I utilize the beliefs and practices surrounding food in the nineteenth-century asylum as a lens through which to juxtapose the intended aims of moral treatment against its implementation. I argue that while the theory of social control continues to provide a useful framework through which to articulate the *intended* functioning of the asylum, a close reading of the historical record provides evidence of the ways in which that intention was thwarted, thereby compromising the ideological effects embedded in the asylum’s design. Proceeding from recent scholarship in historiography, archaeology, and architectural history, I target the “messy slippage between the shape of a place and how it was really used” (Yanni 2007, 14): sites where the orderly, systematic, and efficient functioning of the asylum in theory collided against the disorderly, unpredictable, and often disappointing world of institutional practice. The realm of food was both extensively theorized by administrators seeking to create the ideal asylum and explicitly recounted by patients bearing witness to their confinement. Accordingly, food provides a productive vehicle for engaging the asylum: not simply as a machine for universalizing “bourgeois morality” (Foucault 1971) but as the site of everyday encounters that were formed in the nexus between theory and practice, domination and resistance.

### The role of food in moral treatment

Nineteenth-century psychiatrists assigned food – its production, nutritional content, and the behaviors surrounding its consumption – a major role in the program of moral treatment. In doing so, they drew upon an extensive legacy of ancient and early modern knowledge that offered guidelines for achieving a healthy mind and body. Notably, these guidelines were not purely medical in nature, but made explicit connections between dietary health and moral values. In *The Anatomy of Melancholy*, Robert Burton identified improper diet as a contributor to both physical and mental disorder, claiming that “gluttony” – one of the seven deadly sins – “kills more than the sword” (1621, 196). The link between food and morality would persist into nineteenth-century medicine. In his 1873 treatise *The Ten Laws of Health*, physician J. R. Black traced the entanglement of morals and food to the Bible, attributing mankind’s discovery “that there are such things as good and evil” to the consumption of “forbidden fruits” (1873, 86).

Although they were increasingly disinclined to explicitly invoke the Bible or God, nineteenth-century psychiatrists endorsed essentially the same rules as their early modern predecessors, which they reframed within a biomedical model predicated on “natural” ways of living. As Nature came to stand as a desacralized proxy for the divine within medicine, it assumed a God-like role in arbitrating the rules of the universe and dealing out correspondent rewards and punishments. Individuals who obeyed the rules of Nature would be rewarded with good health, while violators were penalized with illness and infirmity. Samuel B. Woodward, the physician-superintendent of the Worcester State Hospital in Massachusetts, wrote that “transgressions of the laws of life,” such as “eat [ing] excessively … will be followed by summary punishment, as surely as violations of the moral law” (*Annual Report* 1845, 61).

In the context of an industrializing and colonialist society, food and its consumption were inevitably drawn into discourses of progress, culture, and evolution. Here, physicians faced a quandary. The type, preparation, and consumption of food were afforded key roles in defining civilization and distinguishing modern people from the lower orders of humanity. According to Black, “[t]he barbarian takes food and drink as prepared by nature,” while “the man of culture as modified by art” (1873, 83). These postures of gentility were essential to marking Westerners as culturally and intellectually superior – yet physicians believed that the departure from “natural” ways of living exerted pernicious effects on health. Because “barbarians” lived in a state of nature, they were spared the “diseases of civilization.” On the other hand, the “sensualism” and “over-adequateness” exhibited by modern people represented a violation of natural laws (83). Nature, in turn, responded by inscribing the stigmata of this violation onto the offenders’ bodies. Physicians bemoaned the fact that while “savages” and American slaves enjoyed “excellent and beautiful teeth,” refined young women were forced to use dentures (90). Digestive and sexual disorders, intemperance, and insanity were likewise counted among the afflictions that were imposed as punishment for artificiality and indulgence.

Despite their belief that civilization was to blame for much of the disease and debility suffered by modern people, physicians did not suggest that their patients revert to a state of savagery; rather, they recommended education in order to bring modern people back into alignment with the rules of nature while retaining the graces and comforts of society. According to physicians, the privileges of civilization came along with a particular set of temptations that the uneducated masses were poorly equipped to resist. Books such as Black’s *Ten Laws* and M. G. Kellogg’s *Hygienic Family Physician* were targeted at these masses, encouraging the assumption of an ascetic, “natural” diet in defiance of the abundance and artifice of modern society. A meal should consist “of as few articles as possible: bread, meat, one kind of vegetable. Temperance in diet, water for drink, and hard work for exercise will save and prolong life” (Culverwell 1848, 19). Moderation was the law of nature; extremes in either direction were unnatural and should be avoided. The fear of illness and infirmity served to discipline the readers of these texts into conformity with the authors’ “commandments.” The specter of insanity would have presented a particularly powerful incentive to obey the rules of nature. After all, the lunatic suffered not only from the loss of his reason, but also from the stigma surrounding insanity: a stigma that was reinforced by physicians’ assertion that lunatics were transgressors of natural laws.

### The law of moderation

In explaining how diet led to insanity, psychiatrists targeted opposite ends of a social spectrum that was increasingly polarized. On the one hand, they claimed, insanity could be caused by “sensualism” and “over-indulgence,” as exhibited by the upper and middle classes; on the other, it could be caused by the privation and starvation that afflicted the poor. Starvation led to the “deterioration” of the body, which in turn sapped the brain of energy, causing “the manifestations of mind [to] fail [along] with the other functions” (Conolly 1847, 66). Once established, insanity could also work to deaden the appetite, leading to a vicious cycle of worsening mental health. Accordingly, a lack of adequate nutrition was viewed as both a contributor to and a symptom of insanity. The effects of starvation on the mind were so radical that in certain cases the provision of adequate food was thought to be sufficient to improve and even cure mental disease (Griesinger 1866).

The dangers posed to mental health by over-indulgence stemmed from the overstimulation of the senses. Physicians believed that too great a focus on sensory pleasures in the form of “animal food” gave “undue ascendancy to the lowest propensities, leading to aberration and insanity” (Fowler 1856, 27-8). While the pauper insane often suffered from food scarcity, psychiatrists accused them of over-indulgence in drink, a discursive gesture that conveniently shifted blame back onto the individual and away from structural inequalities. According to Black, intemperance was “the most influential of all exciting causes of insanity among the lower classes” (1873, 132). Most American psychiatrists prohibited the recreational use of alcohol in asylums, and were shocked to learn that abstinence was not enforced by their British counterparts. In visiting the Chesire Lunatic Asylum in 1860, Massachusetts psychiatrist Edward Jarvis reported to his wife that beer “being a very common drink here, it is given to patients who need to live well” (Jarvis and Jarvis, 1860, n.p.). The stance against alcohol taken by American psychiatrists can be attributed to their membership in the Protestant, native-born upper middle class, which at the time was experiencing a swell of temperance enthusiasm that openly linked the use of alcohol with immorality, degeneration, and vice. Some psychiatrists, such as Pliny Earle, went a step further in condemning the use of tea and coffee (Earle 1858).

Psychiatrists’ beliefs surrounding the “proper” amount and quality of food, the evils of over-indulgence and intemperance, and the dichotomous opposition of civilization and nature were inflected by their values – a type of “moral synthesis” that functioned to “guarantee bourgeois morality a universality of fact and permit it to be imposed as a law upon all forms of insanity” (Foucault 1971, 259). In presenting these beliefs as objective standards derived from Nature, physicians denied the possibility of legitimate alternatives. For example, while recognizing that individuals exhibited different tastes in food, physicians believed that these differences were learned, not innate, representing deviations from the body’s natural predilections. “Natural taste,” wrote Black, “when left to itself, selects precisely the kind of food suitable for the body” (1873, 93). Taste became corrupted when “dishes [were] made so palatable that the temptation to over-eat is almost irresistible.” Similarly, John Stolz wrote that “sense” was “wonderfully perverted by disobedience to its mandates,” specifically the use of “artificial stimulants, acrid and narcotic substances … Pernicious habits lessen sensibility and destroy the natural relish for healthful food and drink” (1872, 86). Such perversions were not only threatening to health but also to “moral and intellectual development” (Fowler 1856, 27-8).

The theory of violation provided the connecting link between immorality and mental pathology, enabling the inscription of social standards – such as the “laws” of moderation, intemperance, and productivity – into medical etiologies. These rules reflected the social backgrounds of medical practitioners who generally belonged to the upper middle-class and drew upon Calvinist and Quaker value systems that valued a hard-working, self-denying lifestyle. To members of this class, the unhealthy, intemperate, or insane person was guilty of a double offense: violation of the laws of nature as well as the laws of society. As a result of the entanglement of medical knowledge with moral concepts of sin and punishment, psychiatrists were primed to view their patients – and particularly those of the morally inferior lower classes – as offenders rather than victims. This, in turn, influenced whether psychiatrists viewed their patients as worthy or unworthy, curable or incurable.

Although upper- and middle-class patients were also seen as violators of social norms, psychiatrists were more likely to be sympathetic towards these failings and even to see them as valuable. While lower class patients’ illnesses were attributed to their crudeness, ignorance, and laziness, neurotic symptoms in the upper classes were often seen as byproducts of sensitivity, refinement, and intelligence (Scull 2009). Not only were psychiatrists more accepting of the mental afflictions particular to their class, they were also more likely to suffer from them personally or know someone who did. In 1878, John D. Washburn, a trustee of the Worcester State Hospital, suffered from a nervous breakdown that was attributed to the mental strain of his responsibilities as a politician and businessman as well as to “the care and anxiety incidental to the erection of the new hospital for the insane” (“Illness of Col. Washburn” 1878). Ironically, his physician, fellow trustee Dr. Thomas Gage, prescribed a trip to Havana – rather than a stay in the new hospital – as treatment. Likewise Merrick Bemis, the superintendent of Worcester State Hospital, sought treatment for his depressive moods not at his own public institution but at the private Pennsylvania Hospital for the Insane (Tomes 1984).

As Foucault suggests, social class thus played a major role in shaping the diagnosis and treatment of patients in the asylum. Disdain and pity on the part of the psychiatrist toward the patient were inimical to the process of confidence building and mutual respect that was considered essential to moral treatment and contributed to psychiatrists’ belief that the lower classes were less amenable to – and less worthy of – treatment. Even Samuel B. Woodward, who was renowned for his sympathy for his patients, suffered from this prejudice, particularly toward Irish paupers, whose insanity he attributed to “want of forethought … to save earnings for a day of sickness, indulgence of stimulating drinks, and a strong love for their native land.” He complained that it was “difficult” to “obtain the confidence” of these patients, who “seem jealous [suspicious] of our motives.” As a result, “we are not so successful in our treatment of them as with the native population of New England” (*Annual Report* 1847, 33).

### Diet as treatment

If insanity could be caused by improper diet, it seemed logical that treatment would necessitate the substitution of proper dietary habits. Psychiatrists believed that even those patients whose insanity wasn’t caused directly by poor diet could benefit from the consumption of a certain quality and quantity of food. In the first decades of the Worcester State Hospital, administrators spoke proudly of their dietary, claiming that the wholesome and appetizing meals served at the hospital would bolster the physical well-being of their patients, which in turn would promote their return to sanity. Woodward described the hospital diet as “[s]imple and substantial food,” consisting of “meat [at least] once a day … bread of the best quality, vegetables in season, coffee in the morning and tea at night,” and claimed that the menu was “never the same two days in succession” (*Annual Report* 1840, 77).

Because both upper and lower classes of patients were guilty of transgressing nature’s laws, psychiatrists believed that the asylum dietary should be aimed at equalizing both of them, aiming for a state of “natural” moderation. Accordingly, John Conolly advised that “[d]iet should be for the insane pauper more liberal and nutritious than usually found in his cottage,” and “for the wealthier patient simpler and plainer than that usual at his own table” (1847, 70). W. A. F. Browne proposed a system based on the incentivizing of certain varieties of food and other “coveted luxuries” as rewards for labor, arguing that the preferential treatment of certain patients – at least in regards to diet – should be based on merit rather than status (1837, 197). Similarly, George Parkman suggested that the “quantity of food [should be] proportioned to activity,” as an “inducement to employment” (1817, 18). Because upper-class patients were less inclined to work than their lower-class counterparts, a meritocratic system would have mainly benefited the latter.

Woodward claimed that unlike British institutions, which maintained hierarchies of “5-6 classes [of patients], each with different food,” at the Worcester State Hospital “we make little distinction in the ordinary diet, directing from time to time such as particular individuals may require” (*Annual Report* 1843, 70). These requirements were based on medical need, such as “convalescents from mania” who “require substantial food in liberal quantities” in order to replenish their exhausted energies (Woodward n.d.). Other psychiatrists also endorsed the differentiation of diet based on medical need, such as John B. Chapin who advocated for the use of a “special dietary” for those patients who seemed the most likely to recover, in order to maximize their chances of cure (1889, 14).

In practice, however, patients’ social status generally offset other factors in determining their treatment. Most asylums that served both private and pauper patients reserved more diverse and high-quality diets for the former, and the Worcester State Hospital was no exception. While it may have been true that during Woodward’s tenure “little distinction” was made in patients’ diets, by 1902 the trustees – faced with accusations that the food served at the hospital was low quality – were forced to admit that “all private patients paying over $5 have a special diet” (*Annual Report* 1902, 20). Furthermore, upper- and middle-class patients were more likely to benefit from differentiation even when such differences were not directly tied to social rank or wealth. For instance, the privileging of patients based on “curability” tended to favor the middle and upper classes, who were generally viewed as more amenable to cure than their lower-class counterparts.

Classificatory systems based on merit were similarly likely to favor members of the native-born, Protestant middle class – whom psychiatrists viewed as hard-working and industrious – than paupers, who were typed as lazy and ignorant. A patient’s refusal to work was viewed differently depending on his or her social class. While a pauper’s refusal was viewed as evidence of laziness and moral weakness, in the upper class it was viewed as the understandable expression of their refinement and delicacy. Furthermore, upper-class patients didn’t need to work in order to gain access to the special treats offered as rewards to laborers, as they were often supplied with “luxuries” by friends and family members. In her letters to her brother, Mary E. Blanchard described the gifts she brought to their mother, Sarah Seaver, a patient at McLean Asylum in the 1850s, including figs, brandy, strawberries, ice cream, and maple sugar, as well as a specially made cake and candy for her birthday (1851). Patients such as Sarah Seaver would have had a very different culinary experience from those whose families were unable or unwilling to supplement the asylum’s diet with special treats.

### The asylum farm

**Fig. 1:**
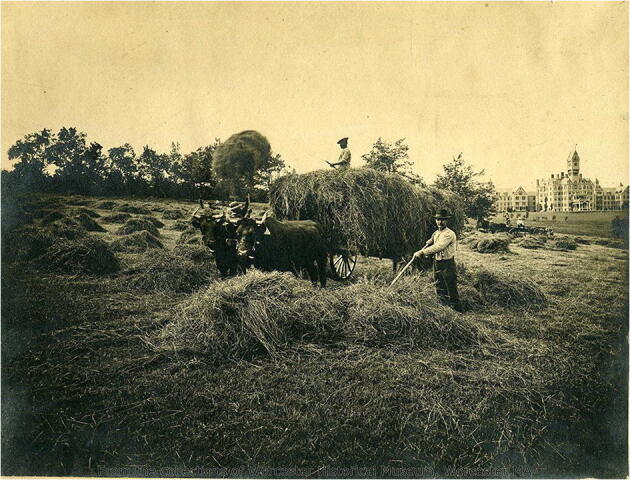
Patients working on the farm of the Worcester State Hospital, circa 1880s-1890s. Courtesy of the Worcester Historical Museum, Worcester, MA

A large portion of the food served at the Worcester State Hospital was grown on its farm, which was worked by patients (Fig. 1). Under this system, patients served as both producers and consumers in a closed circuit that embodied the hospital’s drive toward self-sufficiency – at least in theory. A sequence of patient labor linked the initial planting of produce to its harvesting, preparation, and finally serving. According to administrators, this labor was primarily therapeutic in nature with its contribution to the hospital economy constituting a secondary benefit. For Foucault, the primary utility of asylum labor was to exercise “a constraining power superior to all forms of physical coercion,” serving as yet another entry point for “moral rule” over the patient (1971, 247-8).

Yet the theories and practices surrounding patient labor in the asylum were fraught with inconsistencies. In the 1847 annual report, Woodward attempted to reconcile an apparent contradiction in psychiatric theory: namely, if it was true (as psychiatrists claimed) that “healthful agricultural employment … [was] one of the best restoratives” for the insane, why was it that farmers were the most heavily represented occupation among the male patients in the Worcester State Hospital (*Annual Report* 1838, 4)? Woodward explained that while “moderate outdoor labor” was beneficial, economic demands forced farmers to work “too constantly and severely” for “small remuneration, leading to “over-exertion … to supply the artificial wants created by the present state of society” (*Annual Report* 1847, 42). While the hospital could offer a temporary reprieve, it could not address the systemic problems that caused farmers to overwork in the first place. Woodward didn’t explain what would happen to such patients when they were released and forced to resume their exhausting occupations. According to Pliny Earle’s study of patients discharged from Worcester, many of these individuals suffered relapses that forced them to return to the asylum multiple times (1877).

In order to furnish the means for “moderate outdoor labor,” the first Worcester State Hospital was equipped with a sixty-acre farm (*Annual Report* 1838). Driven by increasing economic needs, the farm was expanded to seventy-five acres in 1845 and to one hundred acres in 1846 (*Annual Report* 1845; 1846). The construction of a second hospital in the 1870s provided the opportunity for an even greater land grab, encompassing 275 acres (*Annual Report* 1871). In the 1890s, administrators constructed a large farmhouse, separate from the main hospital complex, to accommodate the fifty patients who worked at the farm, along with their supervisor, his family, and other hired help (*Annual Report* 1894). The fifty patients selected for this work were described as “reasonably quiet and orderly, able to accomplish a certain amount of work on a farm.” As a reward, they were given “greater freedom” and a “more liberal diet,” which administrators hoped would incentivize good behavior among the other inmates of the hospital. The medical classification of patients thus overlapped with the hospital’s specialization of labor as well as its system of rewards and punishments, reinforcing the connection between mental health, morality, and productivity.

Like the building itself, the second asylum farm quickly became insufficient to meet the demands of its growing population. In the first decade of the twentieth century, administrators secured the purchase of Hillside Farm, a one hundred thirty-acre property in the neighboring town of Shrewsbury, to serve as an auxiliary “farm colony,” providing a site for additional fields as well as accommodations for the hospital’s cows and pigs (*Annual Report* 1912). The effect of Hillside Farm was to segregate the hospital population even further, relegating its patient laborers to a space far beyond the observation of the hospital’s administrators, the company of other patients, and the therapeutic resources of the main hospital complex. Assuming that these resources actually offered curative potential, the removal of these productive but “incurable” patients from the main hospital might have served as a self-fulfilling prophecy, placing chronic patients into circumstances in which they were less likely to subvert the pessimistic prognosis they had been given. At the same time, this separation also served the function of partially liberating patients from one of the asylum’s most iconic and insidious forms of control: surveillance. As Ellen Dwyer writes, patients who were employed as farm laborers at the Willard Asylum for the Chronic Insane in New York “found it easy to circumvent surveillance” (1987, 139). Where the therapeutic mechanisms of the asylum broke down, so too did its coercive properties. These “microgeographies of reduced surveillance” served as openings for the assertion of patient agency (McGeachan 2017, 65; Goffman 1961).

Despite administrators’ best efforts, the ideal of institutional self-sufficiency was ultimately unachievable. Administrators’ commitment to the asylum farm as a self-sustaining source of agricultural produce meant that the hospital was subject to the vicissitudes of the market, the weather, and disease. Proceeds from sales of the hospital’s produce declined during the Civil War, while periodic droughts forced the hospital to temporarily increase its dependence on exterior sources of food, driving up expenditures. In 1915, the hospital’s entire herd of Holstein cattle was infected with hoof and mouth disease, necessitating their slaughter (*Annual Report* 1915). Although patients provided free labor, they required the instruction and oversight of paid supervisors in order to work effectively. Critics charged that the proceeds of asylum farms failed to justify their existence. In 1902, Paul Mange, a farmer in nearby Millbury, wrote to Rockwood Hoar, a trustee of the Worcester State Hospital, citing an annual report that indicated the hospital farm “cost nearly $25,000 to run” while producing “a return of only $4,000” (Mange 1902). Mange speculated that the treasurer had failed to account for the value of the produce that had been consumed at the hospital, such as milk and vegetables, and had recorded as proceeds only the value of items that had been sold on the open market.

Scrutiny surrounding the economy of the hospital farm, and of the hospital in general, increased in the latter half of the nineteenth century when the apparent failure of asylums to realize the promises extended by the “cult of curability” led the public to view their financial demands more critically. State hospitals were, after all, dependent on state revenue for their continued maintenance and were therefore forced to justify their existence – if not by the number of cures, then by their productivity. Having abandoned the curative conceit, the Willard Asylum adopted an institutional model predicated solely on the extraction of labor from its “incurable” inmates (Dwyer 1987). Because they were incurable, Willard’s patient population represented a stable and dependable source of labor. The labor pool at Worcester was more capricious, as it included both acutely ill patients who might recover within months as well as chronic cases who might remain institutionalized for decades. It was this latter group of “incurables” who were tapped to work the hospital farm, which expanded in accordance with the growth of the overall patient population. The hospital’s land holdings peaked in the 1940s when its agricultural properties encompassed five hundred acres. The farm ceased operations in the 1960s, after which much of the property was parceled off and sold.

While David J. Myerson, Worcester’s last superintendent, attributes the end of the asylum farm to the decline in its economic viability, it may also be attributed to the growing concern – which Myerson himself cites – that “the major occupational programs in which patients participated met the needs of the hospital, not the needs of the patient” (1980, 99). The mobilization of patients as unpaid laborers was increasingly viewed by both the public and the psychiatric profession as exploitative and unethical. This shift in attitudes toward patient labor occurred in the context of the emergence of anti-psychotic drugs. While they would be criticized by patients and mental health advocates as yet another “chemical straitjacket” that simply transmuted the coercive effects of the asylum into a different medium, anti-psychotics were widely touted by psychiatrists as offering the chance of meaningful recovery to a subset of patients who had been deemed incurably psychotic (Bynum and Shepherd 1985, 3; Fabris and Aubrecht 2014). The more likely patients were to recover, the less willing the public was to accept their relegation to a lifetime of unpaid drudgery. On a broader scale, the renewed hope of a cure (or at least a viable treatment) for mental illness and the reintegration of patients into the community undermined the perceived necessity for large, isolated residential institutions, which were increasingly seen as retrograde and abusive.

### Forced feeding

While psychiatrists were largely intolerant of social and class difference, they were even more hostile toward differences in perception and understanding that were associated with insanity, viewing these symptoms as expressions of deviance. In his memoir, Clifford Beers attempts to make his delusions legible to the reader, making the case that insanity possesses its own internal logic. As Susannah Wilson writes, “delusional utterances can be read as meaningful when read as metaphorical expressions of real suffering and as strategies to ensure the survival of a self under threat” (LeFrancois et al., 2013, 47). One of Beers’s delusions was the belief that he was being poisoned, a suspicion that seemed to be corroborated by the fact that “[n]one of [his] food [had] its natural flavor.” As a result, Beers refused the medicine that was offered to him in the Connecticut asylum to which he was confined:

To ask a patient in my condition to take a little medicated sugar seemed reasonable. But from my point of view my refusal was justifiable. That innocuous sugar disc to me seemed saturated with the blood of loved ones; and so much as to touch it was to shed their blood. (Beers 1913, 24).

Like the hymns of the hospital chapel – which he perceived as “funeral dirges” – the significance of these medicines was warped through the lens of Beers’s disease, transforming intended agencies of cure into instruments of torture. Psychiatrists’ efforts in mobilizing these agencies foundered not because of their failure to select a sufficiently uplifting hymn or to measure the correct dose of medicine but because they refused to consider the patient’s “point of view,” through which seemingly irrational actions were “justifiable.” After all, according to psychiatrists, taste – along with all other senses – ought to be uniform between individuals as a result of their shared human physiology. If the patient’s sensory perceptions deviated from that objective standard, it was the psychiatrist’s task not to accommodate that deviance, but to discipline it into conformity, “ensuring an ethical continuity between the world of madness and the world of reason” (Foucault 1971, 259).

Even if patients didn’t believe they were being intentionally poisoned, many reported that the medicines they were administered had negative effects. In a letter to trustee Rockwood Hoar, former Worcester State Hospital patient Arthur M. Sanborn stated that “the drugs that were introduced into my food and drink [in the hospital] had a partial effect on me, and raised a fever that ran for several weeks” (1898). Woodward wrote in a letter to Horace Mann that a patient had accused the superintendent of “[giving] him medicine to reduce his thought, in his food” (1833). Although Woodward may not have done so, many psychiatrists endorsed the practice of surreptitiously dosing patients. In his treatise on hysteria, S. Weir Mitchell recommended that physicians whose patients resisted the medicinal use of iron powder should secretly introduce the powder into the patients’ food in order to prove that “iron is not so difficult to take as [the patients] have been led to believe” (1877). Other psychiatrists, such as Browne, objected to what they viewed as a violation of the patient’s trust, claiming that dosing patients without their knowledge or consent gave truth to their paranoid suspicions (1875).

**Fig. 2:**
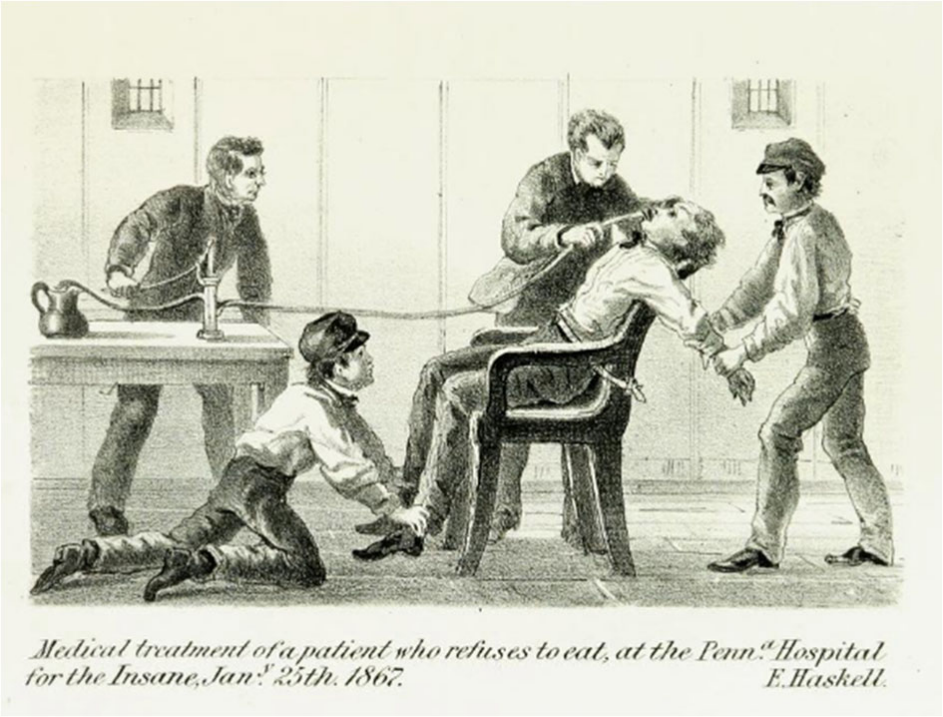
“Medical treatment of a patient who refuses to eat, at the Pennsylvania Hospital for the Insane, January 25^th^, 1867.” From Haskell, Ebenezer. 1869. *The trial of Ebenezer Haskell, in lunacy, and his acquittal before Judge Brewster, in November, 1868.* Philadelphia: Printed by the Author

For many patients, the refusal to eat might offer their only recourse against surreptitious dosing (whether real or imagined) or the severe side effects of medicines as well as their most powerful means of protest against their confinement. Yet psychiatrists uniformly viewed the refusal to eat as a symptom of insanity, and furthermore, a symptom that must be overcome with physical force (Fig. 2). The forced feeding of patients was not without controversy in the psychiatric community. In his 1838 treatise, William Ellis wrote that forced feeding was always unnecessary, as “the patient may be brought to acquiesce by management and skill” (1838). Browne believed that just a small minority of patients necessitated forced feeding, stating that in his experience, “one ninth of the insane refuse food; in [only] one thirtieth of these, the refusal could not be overcome.” Prior to resorting to force, psychiatrists should appeal to “moral and physical means,” such as “bribes, commands, [and] entreaties,” to convince patients to eat. For patients who feared being poisoned, psychiatrists should offer foods that were resistant to tampering (such as whole eggs, fruits, and nuts) (1875, 318).

Over time, psychiatrists’ confidence in their ability to gain the trust and cooperation of the patient declined. Gerald Grob has attributed this tendency partly to the changing patient demographic in asylums, postulating that the influx of immigrants and paupers made it more difficult for psychiatrists to identify with their patients (1966). This lack of a common social footing, along with the belief that these patients were more resistant to treatment and guilty of immoral behavior, may have influenced medical practices and lowered the threshold at which psychiatrists resorted to force. By the mid-nineteenth century, the debate surrounding forced feeding increasingly focused upon when and how the act should be carried out and not whether it represented the most effective or humane solution. In this way, the discourse surrounding forced feeding resembled that surrounding mechanical restraint. While Foucault’s theory of social control is predicated largely on the belief that mechanical restraint had been largely eliminated in nineteenth-century asylums, which instead relied upon the insidious forms of “internalized discipline” imparted by moral treatment in order to control patients, this was typically not the case. In the United States, most asylum administrators characterized the “non-restraint” movement as naively idealistic and oblivious to the realities of managing a large institution (Bynum and Shepherd 1985, 4; Coleborne and MacKinnon 2003, 47). Instead, they advocated minimizing and systematizing – rather than eliminating – restraint in order to ensure it was utilized as humanely as possible.

Criticisms of the asylum often targeted the allegedly abusive use of force against patients. While psychiatrists denied these claims, their reflexive defensiveness suggests that the continuing centrality of physical coercion to asylum discipline was a source of embarrassment to their profession, as it revealed the failure of moral treatment to achieve one of its fundamental aims: to secure the cooperation of the patient by means of gentle persuasion. Yet psychiatrists who used forced feeding believed they had no alternative, as “a medical practitioner who allows a patient to thus commit suicide would deserve the utmost penalties” (Bell 1850, 229). Forced feeding was thus viewed as unavoidable, but it was not without risk. The forced application of a naso-esophageal tube could and did result in the injury and death of patients. Yet even Browne considered the death of a patient as a result of forced feeding a “misadventure” rather than a travesty, justifying the use of the procedure on the grounds that feeding the patient – by any means necessary – was an essential element of treatment, without which the patient would not recover (1875).

Psychiatrists debated over the length of time that should be allowed to elapse before forced feeding was implemented. Like mechanical restraint, forced feeding was regarded – at least in theory – as an instrument of last resort, to be used only when the patient’s survival was in jeopardy. Yet like mechanical restraint, it is evident that in the context of the overburdened, poorly staffed asylum, the expedience of forced feeding made it an appealing alternative to the investment of time and energy that was necessary to earn the trust of an uncooperative patient. Although Browne advised psychiatrists to be conservative in the use of forced feeding, he estimated that the practice had been used in his own asylum “9,000 times” over the course of his twenty-six-year superintendence at the Crichton Royal Asylum (1875, 331).

Furthermore, Browne described forced feeding not only as a form of medical treatment but also as a psychological tactic used to secure the psychiatrist’s dominance, stating that “[a] single introduction [of forced feeding] has often proved sufficient to convince patients of their utter helplessness, of the uselessness of obduracy” (1875, 331). Similarly, Luther Bell stated that “the sight of the syringe may not always fail to be a turning argument” in convincing the patient to eat voluntarily (1850, 230). Some patients, however, refused to yield in spite of the pain and trauma of forced feeding. Browne stated – with a perverse sort of pride – that one patient in the Crichton Asylum had been “supported by artificial alimentation” for more than two years (1875), while Bell reported that several patients at McLean had been force-fed for periods ranging from eighteen to twenty-four months (1850). The procedure that psychiatrists claimed to use only as a last resort in the most extreme situations had thus been ensconced as a part of everyday care for certain patients. Even those patients who were not subject to forced feeding would have become familiar with the procedure through witnessing its use in others: an experience that no doubt shaped their own behavior and emotions surrounding food and discipline in the asylum.

In describing their use of both forced feeding and mechanical restraint, psychiatrists cultivated a narrative of increasingly modern and humane treatment that served to soften the violence and trauma associated with these procedures. In doing so, they obfuscated the fact that many practices used by previous generations in the treatment of the insane – practices that early mental health reformers had decried as obscenely retrograde and barbaric – continued to be implemented in the modern asylum, albeit in slightly altered forms. In an article in the *American Journal of Insanity*, Bell described the instruments of forced feeding used in previous decades, including spouted vessels, iron tunnels, and wooden spoons, which often had the unintended effect of knocking out patients’ teeth (1850). In comparison, Bell presented the use of the naso-esophageal tube as modern and humane, despite the fact that it could — and did — result in injury and death. Bell suggested that his fellow psychiatrists “select the method which *appears* the least coarse and violent” (emphasis original) (230), a curious choice of phrasing that seems to imply that it was more important to maintain the appearance of humane treatment rather than to secure the comfort and safety of the patient.

As subjects of the asylum’s all-encompassing disciplinary regime, patients possessed few effective means of registering their complaints and asserting their agency. Whether motivated by “active delusion” or not, the refusal to eat provided a means by which patients could circumvent the hospital’s control over their bodies and thereby reclaim it for themselves. In describing this refusal as a “case of pure will,” Bell revealed how forced feeding was mobilized as a weapon in the battle between psychiatrist and patient (1850, 227). While psychiatrists may have believed that the patient was delusional, they nonetheless acknowledged that the patient had agency and was capable of exerting it in defiance of institutional dominance. The habitual use of forced feeding suggests that this defiance was seen as more threatening to the institutional order than the delusion itself. After all, delusions were located in the patient’s mind; it was only when they came into active confrontation with the authority of the superintendent that they became problematic and therefore required intervention.

### Tea parties

The practice of dining was conceptualized as an instrument within the asylum’s moral program, which could be used to inculcate certain forms of behavior and even transform character. As Woodward wrote, “[t]he difference between eating food in solitude from a tin or wooden dish with the fingers or a spoon, and going to a neatly furnished table, and taking meals from crockery with a knife and fork, is the difference between a savage and a civilized man” (*Annual Report* 1839, 95). In this statement, “savagery” is coded as insanity; to be civilized is to become sane. Yet the prevailing medical discourse described insanity as a “disease of civilization,” to which savages were virtually immune. According to the psychiatrist, civilization had caused the patient’s insanity, turning a sane man into an irrational savage. To be cured, he must enter the asylum, a “technological marvel” unique to modern civilization (Yanni 2007, 11), where he would eat like a savage, but dine like a gentleman; eschew the pleasures of civilization while meeting its economic needs; return to a state of nature while being educated in bourgeois manners and values. Patients caught in this system could not be blamed for wondering exactly what kind of person the asylum wanted them to be.

The role played by tea in the asylum is representative of this dissonance, occupying a fraught position along the boundary between gentility and vice. Psychiatrists claimed the responsibility for policing this boundary but didn’t always agree on its definition. Pliny Earle believed that the insane should avoid tea and coffee. Similarly, Black wrote that that although “[t]ea, coffee, and tobacco don’t stimulate with the effects of opium and liquors,” they “prepare the way by developing the desire for stimulation” (1873, 124). Woodward disagreed, stating that “tea, coffee, and milk are used liberally in the [Worcester State] Hospital,” with positive results (*Annual Report* 1836, 185). Furthermore, the consumption of tea provided the opportunity to exert a moral influence over the patient. Because the insane were believed to have been reduced to the level of savages, the tea party – and dining in general – acted as an arena in which the patients could practice the arts of gentility. The ability to successfully perform social rituals such as tea drinking was treated as an index of mental health. A sane person demonstrated not only the capacity to enjoy the refinements of civilization but also to maintain self-control, which prevented the pursuit of pleasure from devolving into pathological over-indulgence.

Woodward boasted that at the Worcester State Hospital, “[t]ables were set neatly, furnished with knives, forks, and crockery. We have at no time half a dozen patients who cannot go to the table and eat with knives and forks” (*Annual Report* 1839, 95) – statements that were intended to prove the efficacy of the hospital in civilizing (and thus curing) its patients. He wrote that the majority of patients “conduct[ed] themselves with decorum” at the table (*Annual Report* 1838). Notably, nearly a hundred years later Woodward’s successor, William A. Bryan, would describe the effects of the hospital’s dining program in a similar fashion, writing that in the new cafeteria, “even the most disturbed patients could, after a period of re-education, conduct themselves at the table in a decorous manner” (*Annual Report* 1937, 10). In both instances, the word “decorous” was used as a synonym for “sane” (or at least the appearance of sanity). Bryan proceeded to describe the efforts being made to improve the food service by replacing “heavy crockery” with “dainty china with individual tea pots, tray cloths, and napkins.”

It is illustrative that from its beginning to the 1930s, the therapeutic efficacy of the hospital was couched in terms of teapots, crockery, and the “decorum” that patients displayed in using them. This parallel suggests that little in the institutional standards for recovery had changed over time. Mental health was still largely coterminous with a specific type of social fluency. Even as the new theory and practice of psychoanalysis gained popularity, in the asylum, sanity continued to be articulated in terms of external behavior – i.e., the ability to play one’s part at a tea party – rather than internal thoughts and feelings. Furthermore, the moral program that was intended to instill patients with the “decorum” and restraint appropriate to a tea party represented part of the larger institutional project to render the insane into useful, genteel citizens. Etiquette, along with time-keeping and the production of labor, was one of the many disciplines that drove the ideological machinery of capitalism (Leone and Potter 1999). Through a series of discursive maneuvers that reinscribed the authority of God into that of a scientifically objective “Nature,” these historically contingent codes of behavior were embedded into standards of mental health.

According to Foucault, the objective of institutional discipline was to compel patients to internalize these codes, thus rendering physical force unnecessary. Accordingly, psychiatrists were keen to demonstrate the efficacy of the disciplinary function of the asylum by claiming that the insane were trusted with the use of crockery and even knives. This trust formed a central part of the bonds of “confidence” between psychiatrist and patient that were thought to be essential for recovery (Tomes 1984). Yet psychiatrists’ confidence in the insane was rarely unqualified. As described by Conolly, the knives used by patients at the Hanwell Asylum in England were “only sharpened along a portion of [their] edge,” and the forks had “very short prongs” (Conolly 1847, 75). Accordingly, the objects that were intended to prove the asylum’s trust in its patients may have had the opposite effect, serving as potent reminders of their confinement, as well as signaling the limits of institutional discipline.

Similarly, despite Woodward’s portrayal of perfect “decorum” among his patients, the System of Regulations used during his tenure suggests that meals were heavily choreographed and dependent on the supervision and intervention of attendants: “one Attendant must always be present at the meals, carve the food and distribute it to such as are not competent to do it for themselves, and see that each one has his proper supply. He must also be careful that no knife, fork, or other article, be carried from the table by the patients” (*System of Regulations* 1833, 13). Such regulations speak to the potential for violence that administrators and superintendents tended to downplay in their reports but that represented a daily reality for attendants and patients.

The therapeutic asylum was formed on the premise that the insane, despite their illness, were human beings who were capable of being restored. The modern campaign of moral treatment was traced to Philippe Pinel’s apocryphal removal of the chains from the women at the Salpêtrière Asylum in Paris. The introduction of cutlery and crockery served a similarly discursive purpose in embodying the aims of moral treatment as a humane and humanizing project. It is no coincidence that upon his arrival at the Pennsylvania Hospital as its new superintendent, the first actions taken by Thomas Story Kirkbride were to remove restraints and to construct a “regular dining room” with “ordinary utensils and crockery,” all of which had been “unknown in the old institution” (Tomes 1984, 19). A similar and equally symbolic transformation took place at Hanwell where Conolly gradually replaced metal plates with ceramic pieces, proudly reporting that “breakage is insignificant” (1847, 75). The use of crockery thus came to assume particular significance as a symbol of the humanity of lunatics and the hope for their recovery in the new therapeutic asylum.

It is meaningful that dining etiquette in particular was invested with this symbolic import. In the nineteenth century, “the middle classes adopted ‘table manners’ as a new discriminatory code” (Hamilakis 2014, 23). As a result, “[e]ating lost part of [its] sensuous, experiential value, and became more like a theatre, a performance where one should be constantly conscious of the image one projects” (ibid.). This performance was mobilized in psychiatry as both a means of cure and standard for recovery. Sets of dishes and eating utensils were among those objects that Paul Shackel identifies as “items associated with people who saw themselves as individuals … use to demonstrate merit, punctuality, cleanliness, numeracy, manners, literacy, and other rationalized traits important to modern notions of productivity” (1993, 209). Such values were deeply embedded in discourses surrounding sanity and madness.

Psychiatrists’ descriptions of the “disease of civilization” suggest that the recent rise in the incidence of insanity was threatening not because of lunatics’ personal suffering or the danger they posed to others but due to the loss of their labor in a society that couched human value in terms of productivity. Insanity removed the individual “from the sphere of action and usefulness,” causing “the loss of productive power to families and the State” and “add[ing] to public and private burdens” (“Insanity in Massachusetts” 1861, 94). In 1849, Woodward boasted that as a result of their tenure in the hospital, “[m]any valuable citizens … have been restored to happiness and usefulness in society” (*Annual Report* 1849, 4). For this reason, the ability of the chronic insane to perform “useful” labor in an asylum was viewed as an acceptable outcome when it was acknowledged that not all patients could be cured.

The investment of bourgeois values into standards of mental health meant that not all patients were equally equipped to meet the hospital’s standards, a fact that doubtlessly contributed to the accumulation of “incurables.” Psychiatrists’ perception that the lower classes were less likely to recover was correct, albeit not for the reasons that they claimed. Through no fault of their own, lower-class patients had a distinct experience of asylum life, characterized by a lower quality of accommodations and diet, a greater amount of labor, and less personalized attention from psychiatrists respective to their middle- and upper-class counterparts. Superintendents admitted that they devoted more time and effort toward those patients they viewed as likely to recover: a group that inevitably included more native-born and upper-class patients than paupers and immigrants.

At many asylums, certain patients were invited to dine with the superintendent’s family, a gesture that was intended to build their foundation of trust. According to Morrill Wyman, the son of the first superintendent of the McLean Asylum, his father had “one or more boarders [patients] always at [his] table,” who “had rooms in the mansion house and mingled with the family” (Little 1972, 43). Yet what was presented as an equalizing and progressive gesture was, in fact, a practice that served to further reinforce inequalities. These inequalities might have been less pronounced at private asylums such as McLean, in which most patients belonged to the upper and middle classes. At state hospitals, however, the superintendents’ selection likely contributed to the divide between upper- and middle-class patients and paupers.

During her first few months at the Jacksonville Asylum in Illinois, Elizabeth Packard was among the patients favored by the superintendent’s attention. Along with eating at his table, she was given a well-furnished room, access to her possessions, and the freedom to “read, knit, sew, ride, and walk” as she pleased (1873, 101). Packard would have made an ideal dinner companion for the superintendent: the daughter and wife of ministers, she was well educated and articulate; furthermore, according to the later verdict of the state supreme court, she was not insane. Yet even Packard’s invitation was contingent on her continued deference; after she first openly criticized the institution, her privileges were revoked and she was relocated to the violent ward. “[D]ining with the insane,” she wrote, “I must confess, I did feel more out of my proper place, than I had while in the reception room of refined society” (1873, 81). Like psychiatrists, Packard regarded insanity and refinement as antonyms, conflating social class and mental status.

**Fig. 3:**
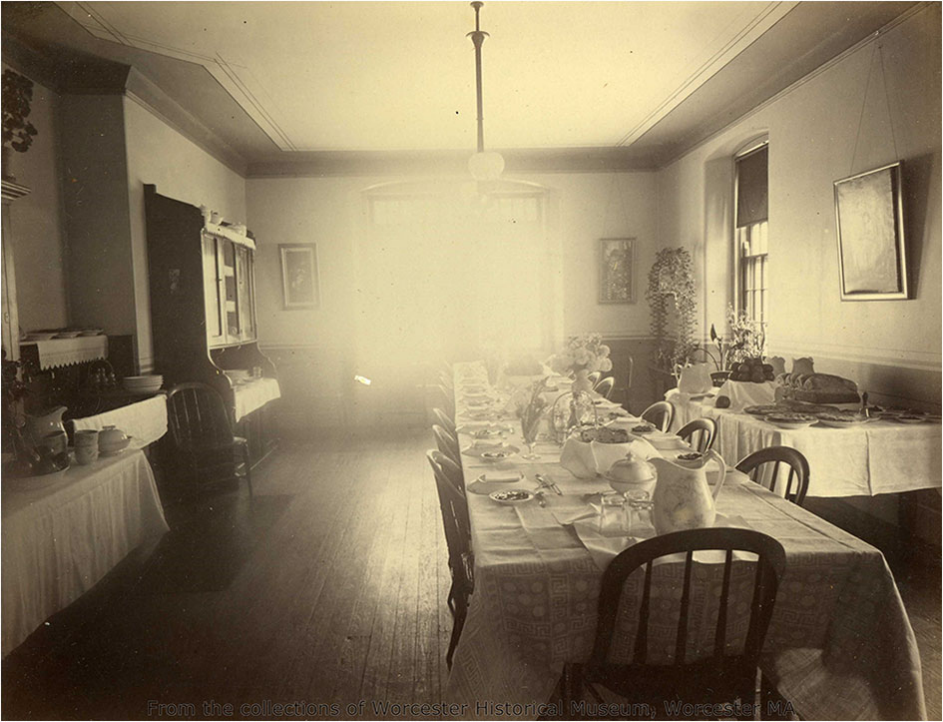
Patients’ dining room, Worcester State Hospital. Courtesy of the Worcester Historical Museum, Worcester, Massachusetts

For those patients who were not invited to the superintendent’s table, dining in the asylum was a decidedly different experience. The patients confined to the Worcester State Hospital’s strong rooms ate without furniture or utensils, their food delivered via an “aperture” in their cells (*Annual Report* 1854, 26). Furthermore, the logistics surrounding dining at Worcester changed radically over time and was heavily impacted by the growth of its patient population. In the first hospital, dining rooms were located in each story of the building, served from external kitchens by dumbwaiters (*Annual Report* 1844) (Fig. 3). In the second hospital, a track running through the hospital basement allowed the distribution of food from a centralized kitchen to dining rooms in each ward (*Annual Report* 1879). Yet these new dining rooms were quickly rendered obsolete by the increasing number of patients and changing standards for dining service. Trustees complained of the difficulty of serving patients in the “numerous small, unattractive dining rooms, many [of which were] distant from the kitchen” (*Annual Report* 1915, 16). By 1904, more than a third of the hospital’s patient population was being “served on trays in the wards” for want of space (*Annual Report* 1904, 14). Later on, tables were crammed into the corridors to meet the need for seating during meals (*Annual Report* 1912).

In the mid 1920s, ward dining rooms were replaced by a cafeteria system (*Annual Report* 1926). While this system was praised by trustees as providing a more efficient means of distributing food, it also eliminated a function that had once been considered an essential part of the hospital’s moral program: the taking of meals in a family style. Writing to Joseph Parish in 1845, Woodward had expressed his preference for small dining rooms over larger ones that mixed residents from multiple wards, stating that the latter compromised classification, exposing “the quiet convalescent patients” to those who were “excited” and “unattuned to habits of cleanliness and order.” “In those small dining rooms those only who are in daily habits of associating come together,” he wrote, which helped promote good feelings and behavior (1845, n.p.). Many of Woodward’s interventions aimed at normalizing the experience of patients in accordance with his own ideas of domestic gentility. This included the taking of meals with known associates of similar comportment and background.

In contrast, administrators in the 1920s justified the use of the cafeteria service on the basis that it gave patients a wider variety of options in menu, claiming that “the responsibility of making choices is of great value in mental rehabilitation” (*Annual Report* 1927, 3). This change in attitude, aside from conveniently justifying an economically motivated decision, reflected changes in theory surrounding the origins of mental illness. To Woodward, the superintendent’s control over every facet of patient dining – from the organization of diners to the type of food and the way it was served – was justified by the fact that insanity was thought to stem from the patient’s poor choices. In order to recover, the patient must temporarily relinquish the power of decision-making to the superintendent who would act as a paternal figure and moral educator until the patient was well enough to exert self-discipline. By the mid-twentieth century, mental illness was increasingly attributed to inheritance and physiological defects that were beyond the patient’s control. Under this model of mental disorder, the significance of dining as a remedial measure declined. While social fluency was still used as an index of recovery and the efficacy of institutional order, the holistic program of moral treatment had been supplanted by an eclectic assortment of therapies – including hydrotherapy, insulin shock therapy, hormone therapy, and lobotomy – that targeted physiology rather than personal discipline as the source of insanity.

### “A cheap imitation of food”

Despite the emphasis that psychiatrists placed on diet as an essential part of their remedial toolkit, the food that was served at state hospitals was widely criticized as nutritionally poor and strikingly unappetizing. Some of the most visceral and repugnant passages in Nelly Bly’s 1887 exposé of conditions at the Blackwell’s Island Hospital are those that describe the food she was forced to consume as a patient. Her first meal consisted of “a bowl of cold tea, a slice of buttered bread and a saucer of oatmeal, with molasses on it.” The butter “was so horrible that one could not eat it”; the tea “had no sugar, and it tasted as if it had been made in copper,” and the bread was “dirty” and “black” in color, “hard, and in places nothing more than dried dough,” and contained a spider. While recognizing her disgust, Bly’s fellow patients urged her to eat, echoing the belief of contemporary psychiatrists that starvation was productive of mental disorder: “To have a good brain the stomach must be cared for” (1887, n.p.). Ironically, this maxim – while embraced by both psychiatrists and patients – was impossible to follow in the asylum due to the poor quality of the food.

Although Pliny Earle – like most psychiatrists – considered good nutrition essential to recovery from insanity, a state investigation in 1880 found that the food served at his institution at Northampton, Massachusetts, was “badly cooked” and “often of bad quality” (“Notes on the Northampton Lunatic Hospital” 1880). Similar complaints against the Worcester State Hospital culminated in 1902, when the poor quality of its food served formed one of the central complaints cited in a widely publicized nurses’ strike (“Nurses in Rebellion” 1902; “Put Out Bag and Baggage” 1902). The nurses claimed that the food served to patients was “rotten and foul-smelling” and that those who refused to eat it were punitively “force fed” – a statement that challenges psychiatrists’ widely held claim that forced feeding was used exclusively as a measure of last resort upon psychotic and delusional patients. If the claims of the nurses, and of Bly, regarding the quality of food served at state hospitals were correct, then patients’ refusal to eat may be reconceptualized as a measure meant to protect their own well-being, and forced feeding as a direct assault against patients’ health and autonomy.

Trustees went to great lengths to repudiate the nurses’ claims, publishing an account of the items that were grown on the hospital’s farm and were thus provided “fresher than what could be obtained at the market,” and obtaining testaments from their outside food suppliers to the quality and freshness of the food. The trustees did concede that the hospital had recently adopted the use of oleomargarine – a newly developed butter substitute that the strikers called a “cheap imitation of food” – due to the prohibitive costs of butter. However, they insisted that the oleomargarine was perfectly edible, and was regularly consumed by administrators and trustees during their visits to the hospital without complaint (*Annual Report* 1902, 19).

Nonetheless, accounts from throughout the early twentieth century corroborate the charge that the food served at the Worcester State Hospital was sub-par. In 1913, the hospital was dogged by another scandal surrounding the alleged waste of food. The hospital’s matron, Mary Dudley, attributed the waste to the fact that patients “sometimes refuse to eat.” Notably, Dudley did not state that the patients’ refusal was a symptom of their insanity; instead, she admitted that many patients “did not like the food,” a grievance that she seemed to take seriously, as she forwarded their complaints to the chef (“Great Waste of Food” 1913). During World War I, administrators strove to increase the institution’s agricultural productivity to compensate for rations imposed on meat, sugar, and wheat (*Annual Report* 1917; 1918). The failure of the hospital’s antiquated refrigerators led to the spoilage of “considerable” amounts of food in the 1920s (*Annual Report* 1924, 3). The Great Depression exerted financial hardships on the institution, leading to shortages of milk, eggs, and fruit despite the continued efforts of the hospital farm (*Annual Report* 1936).

In the 1940s, blood tests showed “low levels of vitamin C’ among patients in the Worcester State Hospital’s back wards, “verg[ing] on clinical scurvy” (Callaway 2007, 5). Along with physical symptoms such as bone pain, easy bruising, and gum disease, scurvy can produce mental symptoms such as mood swings and depression (Léger 2008). In the context of the asylum, where all aberrant behaviors were automatically coded as expressions of insanity, the symptoms of this disease would have been easily misinterpreted as manifestations of the patient’s pre-existing condition. Instead, it is possible that at least part of the pathologies exhibited by patients were iatrogenic: diseases of institutionalization, caused directly by the conditions at the hospital. While originally conceptualized as an instrument of cure, the asylum diet – along with many other facets of treatment – had become a contributor to the very illnesses that it was intended to ameliorate.

## Conclusion

Nineteenth-century psychiatrists ascribed to a model of health that was predicated on the existence of objective and strictly defined laws of nature. To these psychiatrists, illness –including insanity – was a manifestation of the individual’s divergence from these “commandments.” As stated by Woodward:

Insanity is a calculable agency. We see why it befalls and how it may be averted. We see, that should we all obey certain laws, which are annexed to our being, and are the conditions of enjoying mental soundness, we should be exempt from its power; but we also see, that if we will transgress rules, to whose violation the dreadful consequences of insanity have been attached, it is as certain to befall us, as fire is to burn. (*Annual Report* 1838, 6).

The allegedly “natural” rules governing the production of consumption of food, however, were structured by a set of distinctively bourgeois moral values that demonized over-indulgence and intemperance, encouraged self-discipline and productivity, and treated gentility as an index of social worth. Accordingly, the asylum acted not only as a therapeutic instrument but also as a moral machine that was designed to remake lazy, indolent transgressors into useful, “decorous” citizens. Because the theory and mechanics underlying this machine seemed straightforward and self-evident to psychiatrists, they were confounded when the asylum failed to translate its ideals into reality. While psychiatrists tended to blame this failure on the intractable immorality and weakness of individual patients, particularly paupers and immigrants, an analysis of the various meanings and uses of food in the hospital reveals the fault lines that ran through the asylum’s ideological structure.

One of these fault lines can be traced to the dissonances of psychiatric theory, which treated socially and historically contingent values as medical and scientific laws invested with a quasi-divine authority. As a result of their unfailing confidence in the existence of an objective standard of “Nature,” psychiatrists lacked the reflexivity and sensitivity toward difference that were necessary to provide patients from a wide variety of backgrounds with the sympathetic, individualized attention that moral treatment required. Instead of trying to understand patients on their own terms, psychiatrists viewed patients – particularly those who were uncooperative and unrefined – as deviants who needed to be corrected and opponents in a battle of wills. Patients who were faced with the all-encompassing and uncompromising force of the psychiatrist’s authority responded with strategies that were intended to preserve their well-being and autonomy. The resulting dynamic of domination and resistance manifested in the use of coercive, violent, and punitive measures such as forced feeding. Psychiatrists’ myopic focus on insanity as the explanation for any seemingly aberrant behavior prevented them from acknowledging legitimate complaints, respecting their patients’ subjectivities, and questioning their own assumptions. Because the efficacy of the asylum – and, by extension, the psychiatric profession – was thought to hinge on the paternalistic and singular authority of its central authority figure, patients’ acts of resistance were viewed not simply as expressions of insanity, but as challenges to institutional order and to the legitimacy of psychiatry. As his critics have pointed out, the theory of social control as articulated by Foucault largely forecloses any possibility of resistance or expressions of agency on the part of patients (Porter 1985). Yet as the narratives surrounding forced feeding indicate, patient resistance not only existed but represented an ongoing and credible threat to asylum discipline.

A second fault line can be traced to the core identity of the institution, which was founded upon paradoxical concepts of “Nature” and civilization. On the one hand, the nineteenth-century “total institution” was a product of modernity: a “technological marvel” that leveraged the latest discoveries in architecture, biomedicine, and social discipline (Yanni 2007). On the other hand, its therapeutic program was predicated on the reenactment of an idealized, mythologized agrarian past based upon an Edenic vision of Nature (Grob 1966). The result was a Frankensteinian hybrid situated halfway between capitalist machine and feudal kingdom. While intentionally segregated from the world, the asylum was dependent on the nearest city for its population and resources, and upon state appropriations for the maintenance of its infrastructure. While purporting to offer the latest in medical therapeutics, psychiatrists in the asylum were radically segregated from their peers in medicine, which foreclosed their communication and contributed to asylums’ reputation as custodial and scientifically stagnant.

Patients bore the greatest burdens of the hospital’s crisis of identity. They were charged with the work of capitalist laborers, yet denied the incentive of capital; instructed to act like proper citizens while being denied freedom, the defining feature of citizenship; subjected to a program of therapy that treated bourgeois values as irrefutable scientific facts; and expected to recover from insanity while being denied the full advantages of the therapeutic resources that the asylum promised. Their tenure at the hospital was necessary to maintain the stability and specialization of its labor base, yet the hospital’s reputation as a curative institution was reliant on the quick turnover of its patient population. Stuck between its twinned objectives of therapeutic efficacy and economic self-sufficiency, the success of the hospital was predicated simultaneously on patients’ absence and presence. The result of these dissonant forces was the growth of a population of chronically ill but manageable and obedient patients whose existence sustained and justified the survival of the hospital even as it proved the failure of its foundational purpose. It is no wonder that Elizabeth Packard believed that the asylum was a machine whose primary function was the production of “incurables” (1873, 334). In denying patients’ subjectivity, refusing opportunities for reflexive self-criticism, and failing to confront the dissonances and hypocrisies of their discipline, psychiatrists cultivated an environment that was ultimately more productive of pathologies than of cures.

Despite its flawed implementation, the *theory* of moral treatment as articulated by Foucault continues to offer a useful framework for analyzing – at the very least – how the asylum was *intended* to function as “a uniform domain of legislation” (1971: 260). Such an understanding provides a crucial foundation upon which to build a holistic understanding of the asylum as an institution whose theoretical design was dramatically qualified and differentiated in the context of everyday practice. When actualized in the real world – undercut by budgetary restraints, bureaucratic failures, and the vagaries of individual behavior – efforts at practicing moral treatment were not only compromised but often completely nullified. As a result, the subtle and indirect methods of controlling patients that were central to the conceit of moral treatment were rendered ineffective, prompting physicians to resort to physically coercive and violent substitutes. While institutional discipline may have largely prevailed, the violent means by which it was achieved undermine Foucault’s portrayal of the asylum as “a domain of pure morality” and “ethical uniformity” (257). The asylum in practice was characterized by *ad hoc* treatments, moral compromises, and outright repudiations of its foundational principles, including the doctrine of “non-restraint.” For this reason, it is inaccurate to state that “the absence of constraint in the 19^th^-century asylum was not unreason liberated, but madness long since mastered” (252). First of all, constraint never was absent from the 19th-century asylum; secondly, madness was never fully mastered. Administrators’ continuing appeal to violent methods of control was met at every turn by patients’ continuing efforts at resistance.

The war between madness and reason continued to wage throughout the 19th and 20th centuries and into the present day, as mentally ill people’s efforts to register their voices has become increasingly formalized and systematic. The field of Mad Studies represents a popular movement and academic specialty that seeks to radicalize and expand perceptions of madness through the agency of self-identified Mad people (LeFrancois et al., 2013). This movement is largely composed of individuals who have been victimized by psychiatry and its claim as a scientifically objective arbiter of mental health. A study of moral treatment – both as a theory founded upon the mechanisms of “internalized discipline” as described by Foucault and as an ever-shifting and widely variable body of practices situated in particular temporal and geographical contexts – holds the potential to contribute to this movement by historicizing and contextualizing the modern landscape of mental health care. In analyzing the objectives and failures of the first systematic attempt to conceptualize, organize, and cure insanity, scholars may hone a set of analytical tools that can be applied to the present-day medical paradigm. The unwitting investment of social values within the purportedly objective framework of psychiatry, the insidious creep of violence and coercion into the benevolent modality of moral treatment, the unwillingness of physicians to consider the subjectivities of their patients or to hold their own assumptions against the scrutiny of dissenting views, and the relentless efforts of patients to assert the legitimacy of their own perspectives against the crushing devices of asylum discipline all hold momentous implications for the way mental illness is understood and treated today: lessons hard-won by centuries of trial and suffering that should never be forgotten.
